# Engineering heterologous enzyme secretion in *Yarrowia lipolytica*

**DOI:** 10.1186/s12934-022-01863-9

**Published:** 2022-07-04

**Authors:** Weigao Wang, Mark A. Blenner

**Affiliations:** 1 Department of Chemical and Biomolecular Engineering, Clemson University, Clemson, USA; 2 Department of Chemical and Biomolecular Engineering, University of Delaware, Newark, USA

**Keywords:** *Yarrowia lipolytica*, Secretory pathway, Protein production, ER expansion, T4 lysozyme

## Abstract

**Background:**

Eukaryotic cells are often preferred for the production of complex enzymes and biopharmaceuticals due to their ability to form post-translational modifications and inherent quality control system within the endoplasmic reticulum (ER). A non-conventional yeast species, *Yarrowia lipolytica*, has attracted attention due to its high protein secretion capacity and advanced secretory pathway. Common means of improving protein secretion in *Y. lipolytica* include codon optimization, increased gene copy number, inducible expression, and secretory tag engineering. In this study, we develop effective strategies to enhance protein secretion using the model heterologous enzyme T4 lysozyme.

**Results:**

By engineering the commonly used native lip2prepro secretion signal, we have successfully improved secreted T4 lysozyme titer by 17-fold. Similar improvements were measured for other heterologous proteins, including hrGFP and $\alpha$-amylase. In addition to secretion tag engineering, we engineered the secretory pathway by expanding the ER and co-expressing heterologous enzymes in the secretion tag processing pathway, resulting in combined 50-fold improvement in T4 lysozyme secretion.

**Conclusions:**

Overall, our combined strategies not only proved effective in improving the protein production in *Yarrowia lipolytica*, but also hint the possible existence of a different mechanism of secretion regulation in ER and Golgi body in this non-conventional yeast.

**Supplementary Information:**

The online version contains supplementary material available at 10.1186/s12934-022-01863-9.

## Introduction

As a dimorphic oleaginous yeast, *Yarrowia lipolytica* has been found widely existing in soils, sea water and different kinds of food [1]. It is generally regarded as safe (GRAS) by the Food and Drug Administration (FDA) [2], motivating its use in a wide range of industrial applications, including heterologous protein production, bioremediation, and value-added metabolites [3–7]. Interest in the application of this yeast prompted the development of myriad synthetic biology tools [8–12]. To promote *Y. lipolytica* as a host for protein secretion, strains that have alkaline and acidic extracellular protease have been knocked out and non-reverting auxotrophies have been established [12, 13].

*Yarrowia lipolytica* is touted as an extraordinary host for protein production based on its secretory machinery being more complex than the model yeast, *Saccharomyces cerevisiae,* with higher similarity to mammalian cells than to yeast, e.g. preferential use of co-translational translocation [14], and the large capacity for the production of extracellular proteins [9, 15–17]. Even though most of the possible individual elements of the secretory machinery have been identified by either proteomics or comparative genomics approaches [15, 17], most common strategies to enhance the protein production in *Y. lipolytica* mainly focus on codon optimization, increasing the gene copy number, regulatory elements engineering and optimization of culturing conditions [18, 19]. Engineering the secretory pathway to improve the protein production in *Y. lipolytica* hasn’t attracted as much attention as in *Saccharomyces cerevisiae*. In addition to codon optimization, studies to improve protein secretion in *Y. lipolytica* have focused on global transcriptome profiling, identification of signal peptides, and secretory helper co-expression [20–22]*.*

Most of the heterologously expressed secreted proteins expressed in *Y. lipolytica* are fused with native secretion signals xpr2prepro and lip2prepro, or their hybrid sequences [1, 9, 18, 23]. Lip2prepro is a native secretion signal found on the extracellular lipase 2 gene, *LIP2*. While directing translated peptides into the endoplasmic reticulum (ER) from the cytosol, the dipeptide (X-A/X-P) stretch of the secretion signal is recognized and processed by aminopeptidase in the ER. When the peptide is transported from the ER to the Golgi, the pro sequence is cleaved off to produce mature protein [23]. In the secretory pathway, the ER and Golgi play a key role in protein folding, post-translation modification, and quality control of translated protein. Deleting the phosphatidic acid phosphatase gene, *PAH1*, impaired triacylglycerol formation and sterol ester storage, and triggered proliferation of the ER membrane in *Y. lipolytica* [24, 25]. The same strategy was successfully used to enhance protein secretion in *S. cerevisiae* [26]. Other approaches for improving protein secretion in *S. cerevisiae* include overexpressing ER exit chaperone *ERV29*, which improves the ER exit efficiency [26].

In this study, secretion of T4 lysozyme was used to evaluate strategies to increase protein secretion. By optimizing the combination of a native secretion signal lip2prepro and intron sequence, we have successfully improved extracellular titer of T4 lysozyme by 17-fold. We demonstrate the generality of the combination optimization between the secretion leader sequence and intron sequence by showing enhanced secretion of hrGFP and α-amylase. To further improve the secretion yield, we co-expressed important enzyme in the ER and Golgi body with the knockout of *pah1* gene in ER, showing that overexpression of scEVR29 in a Δpah1 strain with the lip2pre-intron signal resulted in 50-fold improvement of T4-lysozyme secretion.

## Materials and methods

### Chemicals and reagents

Growth medium components were purchased from Difco Laboratories. Q5 polymerase chain reaction (PCR) components (Catalog number: M0419S) and T4 Polymerase (Catalog number: M0203S) were obtained from New England Biolabs^®^. All chemicals used for buffers were purchased from Sigma-Aldrich, unless stated otherwise. SDS-PAGE gels (Catalog number: 4561096) and Precision Plus Protein All Blue Prestained Protein Standards (Catalog number: 1610373) were obtained from Bio-Rad. Goat anti-rabbit IgG Polyclonal Antibody (IRDye^®^ 800CW, Catalog number: 925-32,211) was purchased from LI-COR Biosciences. HIS tag (H20) Antibody (Catalog number: DB063) was purchased from Delta Biolabs. Nitrocellulose membranes (Catalog number: 10600002) were purchased from GE Healthcare Amersham™. gBlock™ Gene Fragments and oligos were synthesized by Genscript Biotech and IDT DNA, respectively. Miniprep (Catalog number: 76358-832) and DNA Clean and Concentrate (D4004) kits were purchased from Zymo Research.

### Strains, primers, and plasmid constructions

DH10β cells (NEB Catalog number: C3019H) were used for cloning and propagation of plasmids in Luria Bertani (LB) media supplemented with 100 μg/mL ampicillin. *E. coli* cells were transformed using a standard heat shock method [8]*. Y. lipolytica* strain PO1f (*MATa leu2-270 ura3-302 xpr2-322 axp1*) (ATCC no. MYA-2613TM) was used for protein expression studies. Primers used for vector construction in this study are summarized in Additional file 1: Table S1. pSL16-pTEF + intron vector was amplified from a previously constructed plasmid containing tef promoter, 122-bp tef intron fragment and CYC terminator [8]. pSL16-pTEF+intron vector was ligated with T4 lysozyme gBlock fragment containing lip2prepro sequence by Sequence and Ligation Independent Cloning (SLIC) method to produce pSL16-pTEF+intron -lip2prepro-T4 lysozyme [27]. pSL16-pTEF+intron -T4 lysozyme, pSL16-pTEF+intron -lip2pre-T4 lysozyme and pSL16-tef-lip2pre-T4 lysozyme were derived from plasmid pSL16-pTEF + intron -lip2prepro-T4 lysozyme using exclusion primers listed in Additional file 1: Table S1. Plasmids pSL16-pTEF+intron -lip2prepro-GFP, pSL16-pTEF+intron -lip2pre-GFP, pSL16-pTEF+intron -lip2prepro-$\alpha$-amylase and pSL16-pTEF+intron -lip2pre-$\alpha$-amylase were constructed by replacing T4 lysozyme gene in plasmids pSL16-pTEF+intron -lip2pre-T4 lysozyme and pSL16-pTEF+intron -lip2prepro-T4 lysozyme using the primers in Additional file 1: Table S1.

To construct Ste13 coexpression plasmids, *STE13* gene (diaminopeptidase) was amplified from the genome of *Saccharomyces cerevisiae* with the inclusion of an ER retention signal RDEL. Two plasmids (pSL16-pTEF+intron -ste13 and pSL16-tef(star)-ste13) with different expression strength were constructed and their expression cassettes were PCR amplified and ligated with plasmid pSL16-pTEF+intron -lip2pre-T4 lysozyme. All PCR amplifications were performed using Q5 polymerase (NEB Catalog number: M0491). PCR products were purified using DNA Clean and Concentrate kit (Zymo Research). Ligations were performed by using Sequence and Ligation Independent Cloning (SLIC) described above. The his-tagged hrGFP gene was PCR amplified from UAS1B8-TEF-hrGFP-histag [8]. T4 lysozyme and α-amylase genes were codon optimized for *Y. lipolytica* strain and synthesized with 6x-Histidine tag by Genscript. All synthesized gene sequences are provided in Additional file 1: Table S2.

### Media and cell growth conditions

*E. coli* cells were grown in liquid LB media (1% tryptone, 1% sodium chloride and 0.5% yeast extract) and cultured in 37 °C shaker with speed at 250 rpm overnight. *Y. lipolytica* cells were cultured in 50 mL liquid YSC-LEU media (2% glucose, 0.1 M sodium phosphate buffer (pH 7.0), 6.7 g/L YNB without amino acids (Difco), 0.69 g/L CSM-LEU (MP Biomedicals) in 250 mL baffled flasks at 28 °C and 215 rpm for 48 h. Agar plates were prepared by adding 1.5% agar to either of the above liquid media. Standard concentrations of antibiotics were added where necessary [28].

### Generation and verification of *PAH1* knockout strain

To construct the CRISPR knockout vectors, NsiI (Catalog number: R0127S, NEB) was used to digest parent pCas9-Leu or pCas9-URA plasmids [29]. Corresponding primers were used to produce inserts by the gradient annealing method [29]. The NsiI linearized vectors and annealed products were purified and assembled using SLIC [29]. After confirmation by Sanger sequencing, 1 μg CRISPR knockout plasmids were transformed into the *Y. lipolytica* PO1f strain using the one-step transformation method described previously [30]. Transformants were screened by colony PCR and positive colonies were confirmed by Sanger sequencing. CRISPR knockout vector in *PAH1* knockout colony was then cured by culturing without selection on YPD agar plates (20 g/L dextrose, 20 g/L Peptone, 10 g/L yeast extract and 1.5% agar).

### Preparation of cellular and secreted proteins

*Y. lipolytica* strains were cultivated in 50 mL YSC-Leu + 2% glucose media as described above, for 48 h in biological triplicate. The OD600 values were measured and normalized to an OD600 of 26 for each flask. 40 mL OD600 normalized cultures were centrifuged at 3500 rpm and room temperature. Supernatants were collected and concentrated 40-fold using an Amicon Ultra-15 Centrifugal Filter Unit with a 10 kDa molecular weight cutoff (MWCO) value by centrifuging at 3500 rpm and 4 °C. Concentrated supernatant, containing soluble secreted protein, was stored at 4 °C until use.

Separately, collected cell pellets were resuspended in 5 mL 20 mM sodium phosphate buffer (pH 7.0). 1 mL resuspended cells were added into 2 mL bead milling tube with one scoop of 0.5 mm diameter glass beads (~ 0.5 g). Bead milling was performed using a Biospec-Brand Bead Beater for three cycles with 3 min at 4 °C for each cycle to guarantee complete lysis. Cell lysates were spun down at 12,000 × g for 2 min, and the supernatant was collected. The supernatant, containing soluble intracellular proteins, were stored at 4 °C until use.

### SDS-PAGE and immunoblot analysis

Prepared intracellular and extracellular protein fractions described above were separated by SDS-PAGE using 4–20% Mini-PROTEAN^®^ TGX™ Precast Protein gel (Bio Rad) according to manufacturer protocol. After separation, the proteins were transferred onto a nitrocellulose membrane using a wet transfer method [31]. Membranes were blocked in 5% Bovine Serum Albumin (BSA) solution for 30 min at room temperature, and then incubated in primary antibody solution (1:250 dilution of H20 antibody, 0.5% sodium azide and 2% nonfat milk in TBST buffer (20 mM Tris, 150 mM NaCl, 0.1% (v/v) Tween 20) overnight at 4 °C. Membranes were washed 3 times in 20 mL TBST buffer for 5 min, and then incubated in 20 mL secondary antibody solution (5% BSA, 1:20,000 dilution of Goat anti-rabbit IgG Polyclonal Antibody in TBST buffer) in the dark at room temperature for 1 h. The secondary antibody solution was washed 3 times from the membrane by shaking with 20 mL TBST buffer for 5 min per wash. Membranes were imaged on LI-COR Odyssey CLx imaging system.

### Enzyme linked immunosorbent assay (ELISA)

Black 96-well immunno plates (Catalog number: 12–566-24, Thermo Fisher Scientific) were incubated with 200 µL prepared intracellular and extracellular samples buffer-exchanged with coating buffer (10 mM phosphate buffer (pH 7.4), 150 mM NaCl, 0.1% sodium azide) at 37 °C for 30 min. The plate was washed with 200 µL PBST (10 mM phosphate buffer (pH 7.4), 150 mM NaCl, 0.1% sodium azide, 0.05% Tween20) three times. 200 µL ELISA blocking buffer (50 mM Tris–HCl (pH 8.0), 1% BSA) was added to each well and incubated at room temperature for 30 min. After, the plate was washed with PBST buffer three times. 200 µL primary antibody (Goat anti-rabbit IgG Polyclonal Antibody, Delta Biolabs) in blocking buffer (v/v 1:100) was added to each well, and the plate was incubated at room temperature for 2 h. The plate was washed with PBST buffer as before. 200 µL secondary antibody (Goat anti-rabbit IgG antibody, HRP conjugate) diluted in ELISA blocking buffer (1:3000 v/v) was added to each well, and the plate was incubated for 2 h at room temperature. The plate was washed again with 200 µL PBST three times. 200 µL ECL substrate (Bio-Rad) was added to each well, and the plate was incubated in the dark for 30 min. Chemiluminescence was measured using plate reader (BioTek Synergy Mx).

### Enzyme yield calculation

Pure his-tagged T4 lysozyme was used as the standard to find the correlation between the protein concentration and the chemiluminescent signal intensity in ELISA. The measured chemiluminescence signal intensities of different samples were converted to the protein concentration in $\mu$ g/L and then divided by the measured OD600 values to produce a specific secretion yield in $\mu$ g/L/ OD600.

### mRNA level measurements by Quantitative Reverse Transcription PCR (qRT-PCR)

*Yarrowia lipolytica* transformants grown in 50 mL YSC-Leu + 2% Glucose liquid media for 48 h. Cell culture OD600 was normalized to 10, and 1 mL was centrifuged at 2000 g for 2 min at room temperature. Total RNA was extracted immediately using Qiagen RNA Yeast kit (Catalog number 74140, Qiagen). RNA extractions were placed at −80 °C until further use. RNA, qPCR primers, and Quanta Biosciences qSCRIPT One-Step Sybr Green RT-qPCR master mix were mixed according to manufacturer protocol, loaded into the qPCR plate (Catalog number: HSP9601, Bio-Rad) and sealed with heat-resistant film (Catalog number 60941-078, VWR). All the qPCR primers are listed in Additional file 1: Table S1 and have efficiency > 95%. qRT-PCR was performed by following the standard protocol from Quanta Biosciences using the CFX Connect Real-Time (Bio-Rad). The quantification cycle (Cq) value of each construct was normalized with the Cq value of housekeeping gene $\beta$-actin [32]. The gene expression level change between different constructs was calculated in 2^(−∆Cq)^. All amplification results were checked with melt curves to ensure single products.

## Results

### Optimization of the combination between intron and lip2prepro improves the secretion of protein

**Fig. 1:**
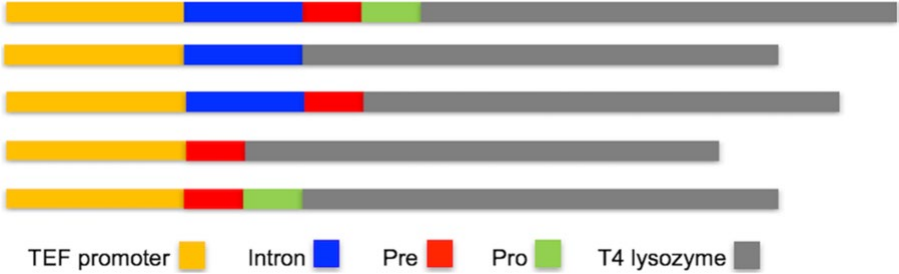
Schematic design of constructs with different combination of intron, lip2pre and lip2pro fragments

**Fig. 2:**
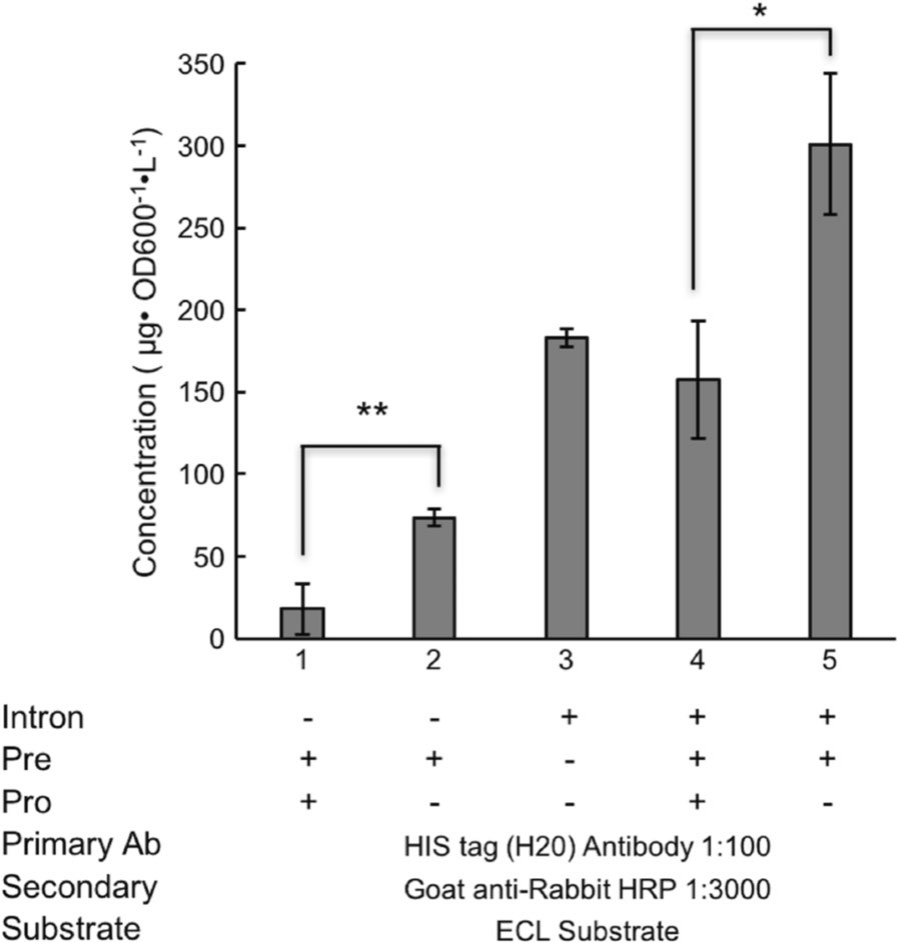
Optimization of the combination between intron and lip2prepro for the improvement on the secretion of T4 lysozyme. T4 lysozyme expression was performed under the control of TEF promoter with different constructs in shake flasks. Changes were statistically compared between with and without pro constructs (column 1 and 2, 4 and 5). *, *P* < 0.05; **, *P* < 0.01. Measurements were reported as the average values $\pm$ standard deviations from individual triplicates

**Fig. 3:**
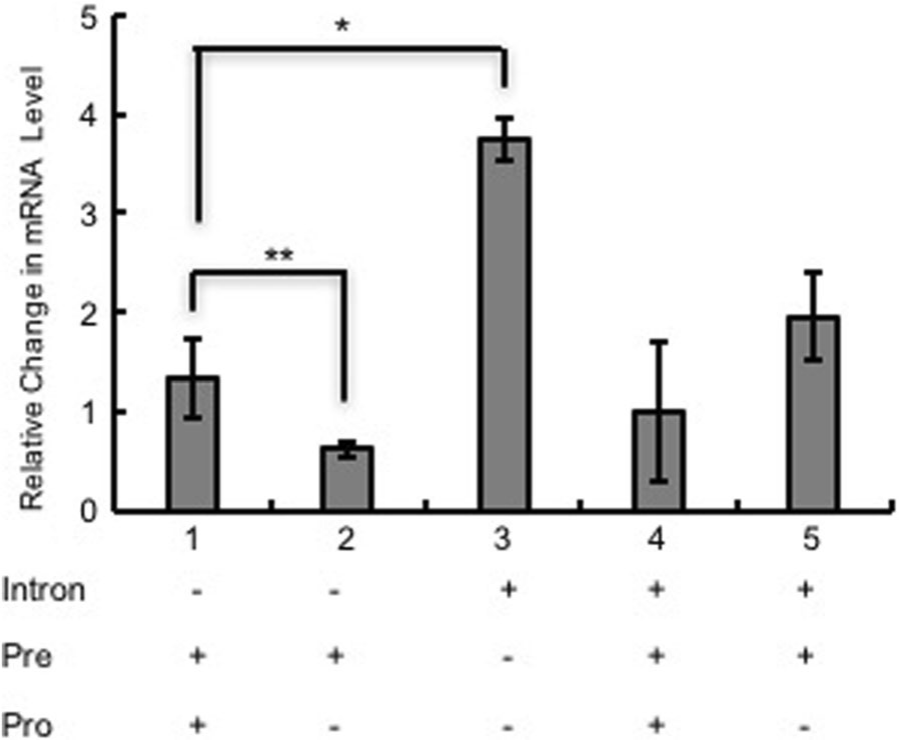
Comparison of mRNA levels of T4 lysozyme from different constructs. mRNA levels are represented as copy number relative to the copy number of the housekeeping gene $\beta$-actin. The expression was normalized using the ∆∆C_T_ method. Changes were statistically compared between with and without pro constructs (column 1 and 2 and 3). *, *P* < 0.05; **, *P* < 0.01; ns, P value non-significant. Measurements were reported as the average values $\pm$ standard deviations from individual triplicates

In our study, we first examined the impact of combining components of the TEF intron sequence with the native secretion signal lip2prepro (Fig. 1). The specific titer of the secreted T4 lysozyme was 18.20 $\mu$ g/L/OD600 with the TEF promoter followed by native lip2prepro secretion signal (Fig. 2, column 1). Removal of the pro sequence from native lip2prepro secretion signal leads to a fourfold increase of secreted T4 lysozyme reaching 73.5 $\mu$ g/L/OD600 (Fig. 2, column 2). Interestingly, this increase was accompanied by a 50% reduction in the mRNA level of T4 lysozyme suggesting the presence of the lip2pro sequence promotes more efficient mRNA production while frustrating secretion (Fig. 3, columns 1 and 2). One may speculate that the pro sequence could unnecessarily prolong the retention of protein in the Golgi and decrease the secretion level. Further replacement of the full lip2prepro secretion signal with pTEF+intron sequence, in which no secretion signal is included, surprisingly resulted in further improvement of T4 lysozyme specific titer to 183.02 $\mu$ g/L/OD600 (Fig. 2, column 3). qPCR analysis of relative mRNA levels revealed a fourfold higher mRNA expression level (Fig. 3, column 3). Given the benefits of higher expression conferred by the pTEF+intron, we sought to learn if a stronger promoter could be combined with the secretion signal for even higher enzyme secretion. Inclusion of lip2prepro secretion signal into the pTEF+intron construct didn’t lead to further increase of the T4 lysozyme secretion (Fig. 2, column 4), while the mRNA level was once again reduced (Fig. 3, column 4)). This suggests that increased mRNA level associated with the pTEF + intron promoter was counterbalanced by the decrease in mRNA level associated with the lip2prepro sequence. Removal of the pro sequence from the secretion signal, once again, increased the specific titer of T4 lysozyme to 300.78 $\mu$ g/L/OD600 (Fig. 2, column 5), and did not lead to significant change in mRNA level (Fig. 3, columns 4 and 5).

**Fig. 4:**
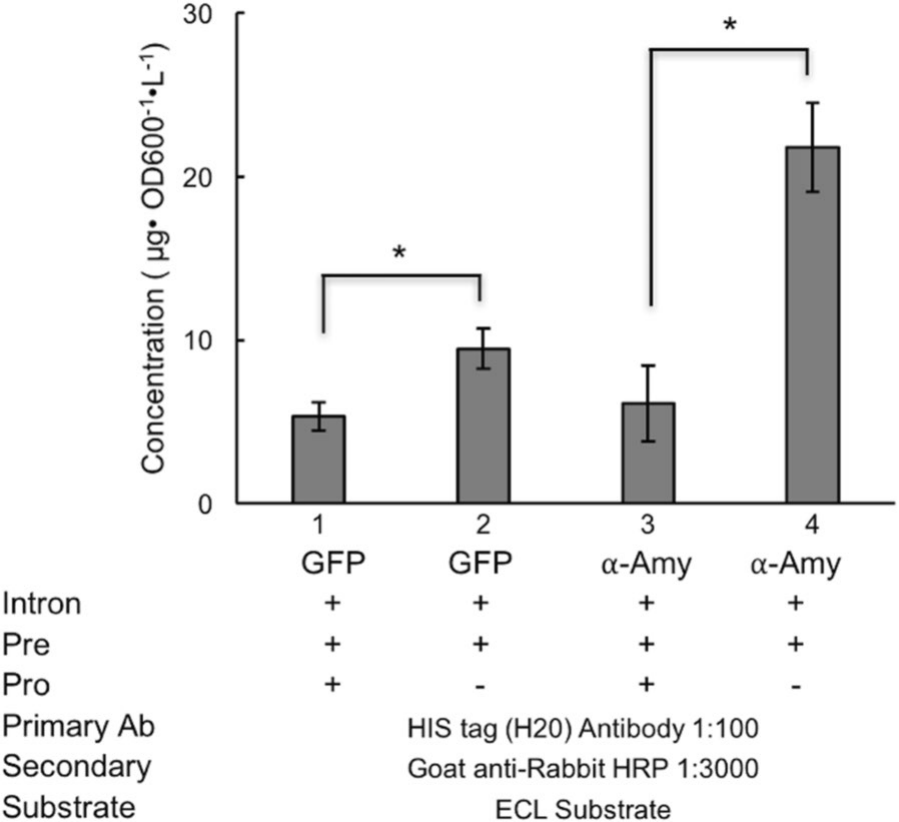
Removal of pro sequence promotes the secretion of GFP and $\alpha$-Amylase. Both the expression of GFP and $\alpha$-Amylase was performed under the control of TEF promoter with or without pro sequence in shake flasks. Changes were measured with comparisons to pro sequence including constructs (column 1 and 3). *, *P* < 0.05; **, *P* < 0.01; Measurements were reported as the average values $\pm$ standard deviations from individual triplicates

The generality of the pro sequence detriment on the protein secretion was further investigated using GFP and $\alpha$-amylase reporter proteins. The specific titers of both proteins with pTEF+intron -lip2prepro were less than 10 $\mu$ g/L/OD600 (Fig. 4). The secretion level of GFP and $\alpha$-amylase showed consistent increase upon the removal of the pro sequence from the pTEF+intron -lip2prepro construct. The secretion level of GFP almost doubled when the pro sequence was deleted. At the same time, the secretion level of $\alpha$-amylase showed fourfold increase upon pro sequence deletion. In summary, the secretion level of T4 lysozyme was improved by around 17-fold after optimizing the combination of intron sequence and secretion signal sequence, and the propeptide was always detrimental. Therefore, we have removed the pro- sequence from our secretion promoters moving forward.

### Co-expression of *scSTE13* and *scERV29* in *PAH1*$\Delta$ strain improve the secretion level

**Fig. 5:**
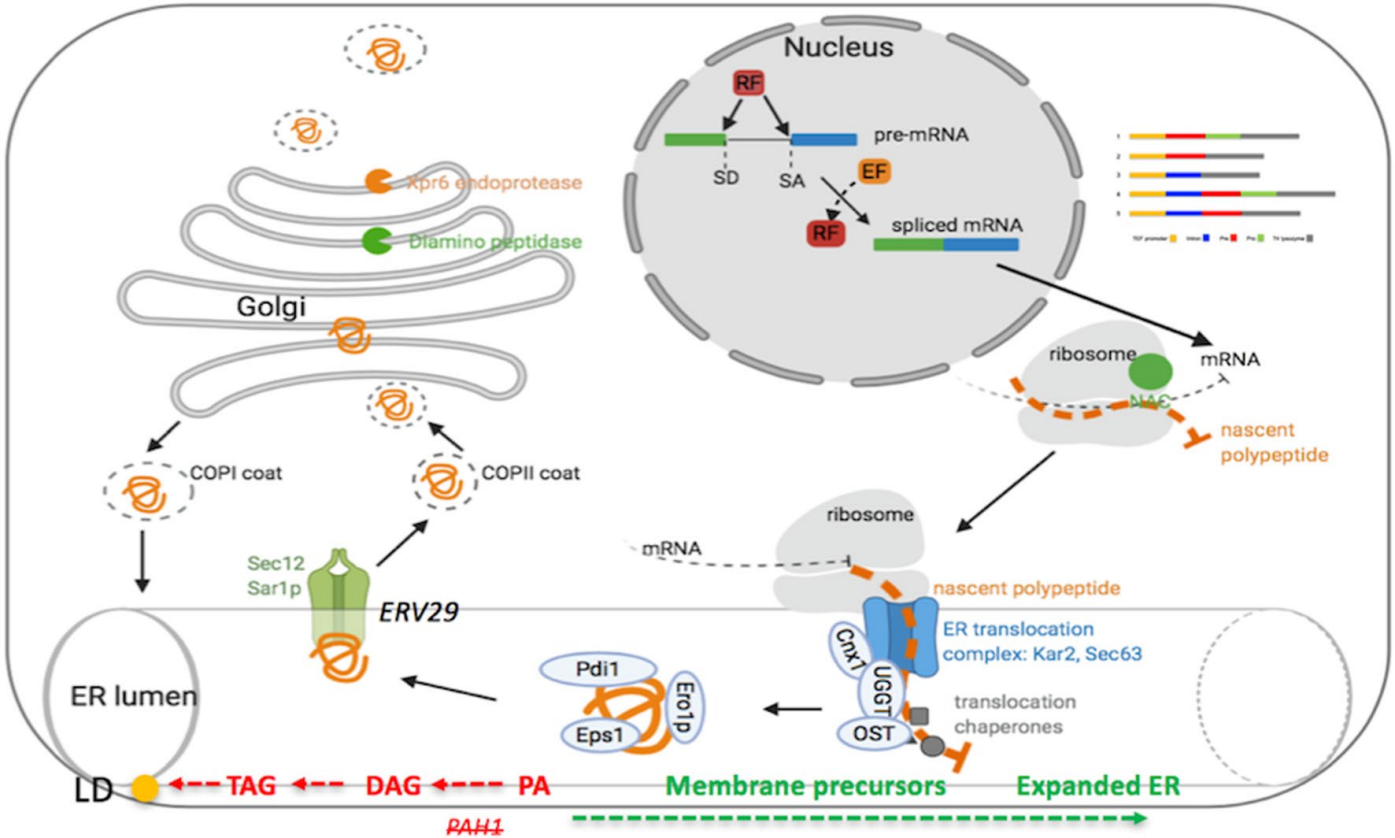
Overview of the strategies applied in the secretory pathway engineering [9, 24, 26]. Exported mRNA from nucleus was co-translated and translocated into ER. The ER membrane was engineered by deleting *PAH1* gene and direct the phospholipid acid for the synthesis of ER. PA, phospholipid acid; DAG, diacylglycerol; TAG, triacylglycerol; LD, lipid droplet; *ERV29*, ER exit receptor

**Fig. 6:**
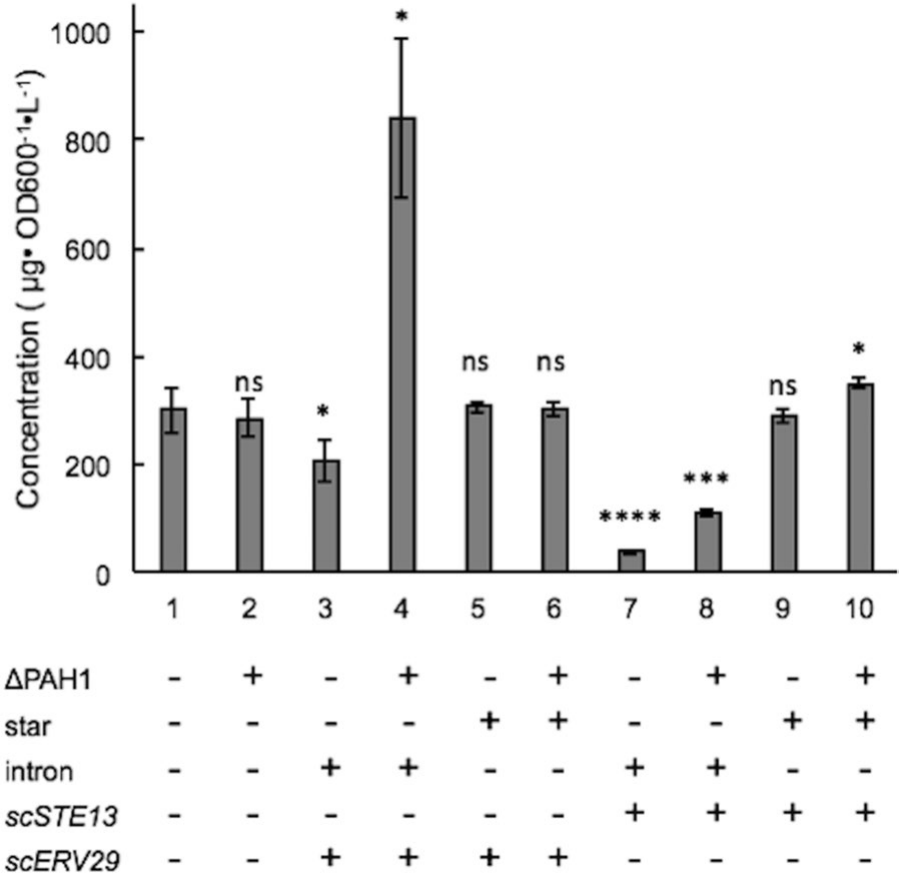
Overexpression of *scERV29* and *scSTE13* in *pah1* $\Delta$ strain affect the secretion of T4 lysozyme. Overexpression of *scERV29* and *scSTE13* were performed under both strong (with intron) and weak (with star) promoters with the combination of the optimized construct. Changes were measured with comparisons to the optimized construct (column 1). *, *P* < 0.05; **, *P* < 0.01; ns, non-significant. Measurements were reported as the average values $\pm$ standard deviations from individual triplicates

**Fig. 7:**
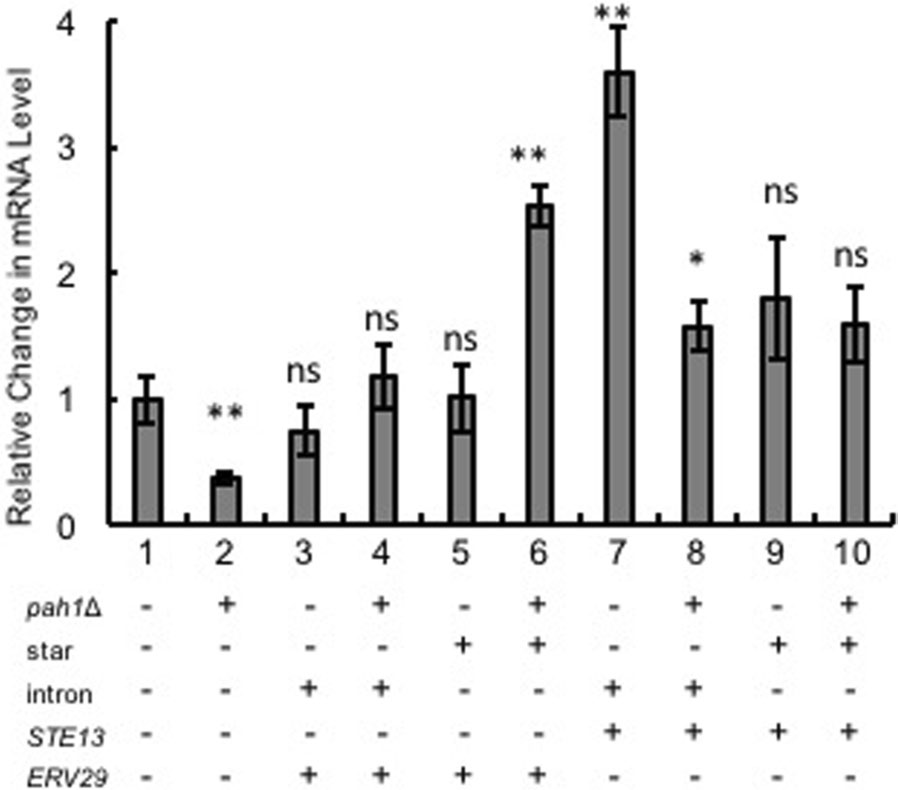
Comparison of mRNA levels of T4 lysozyme from different constructs. mRNA levels are represented as copy number relative to the copy number of the housekeeping gene $\beta$-actin. The expression was normalized using the ∆∆C_T_ method. All changes were measured with the comparison to the optimized construct (column 1). *, *P* < 0.05; **, *P* < 0.01; ns, P value non-significant. Measurements were reported as the average values $\pm$ standard deviations from individual triplicates

Since endoplasmic reticulum (ER) and the Golgi body are two important organelles for protein synthesis and secretion (Fig. 5), we targeted both the ER and Golgi to improve the secretion capability of *Y. lipolytica*. Phosphatidic acid phosphatase encoded by *PAH1* is responsible for the synthesis of the diacylglycerol (DAG) of the storage lipid TAG. According to Guerfal et al. [24], knockout of *PAH1* will disrupt the TAG synthesis pathway and direct the fatty acid flux to phospholipids, leading to ER membrane proliferation. This ER expansion is expected to enhance the membrane protein accumulation levels [24–26, 33]. We hypothesized that an expanded ER would increase the processing efficiency of the secreted proteins and thus improve the secretion of T4 lysozyme. To test our hypothesis, the optimized construct pTEF+intron -lip2pre-T4 lysozyme was transformed into *Y. lipolytica* po1f and *pah1* $\Delta$ strains separately and their secretion levels of T4 lysozyme were compared. As shown in Fig. 6 (columns 1 and 2), knockout of *PAH1* gene does not lead to significant change in the secretion level. Meanwhile, mRNA quantification revealed that the expression level of the T4 lysozyme was decreased significantly by more than 50% when expressed in *pah1* $\Delta$ strain (Fig. 7, columns 1 and 2). Combined, these results indicate that ER expansion did not improve the T4 lysozyme secretion titer, but may lead to more efficient protein section, since the same secretion titers were achieved with lower T4 lysozyme mRNA level.

*ERV29* encodes the ER exit receptor and is important for packing the secretory proteins into COPII vesicles [17]. Disruption of *ERV29* in *S. cerevisiae* (*scERV29*) was reported to enhance the secretion level of $\alpha$-amylase [26, 34]. Since the effect of *ERV29* on protein secretion in *S. cerevisiae* is understood but has not been tested in *Y. lipolytica*, we overexpressed *scERV29* under both weak and strong promoters with the optimized construct pTEF+intron -lip2pre-T4 lysozyme. Surprisingly, the secretion level of T4 lysozyme was reduced significantly to 206.71 $\mu$ g/L/OD600 when expressed under a strong promoter in po1f strain (Fig. 6, column 3, *P* value of < 0.05). This drop in specific titer was unexpected based on results from *S. cerevisiae* [26]. However, the secretion level of T4 lysozyme nearly quadrupled to 842.48 $\mu$ g/L/OD600 when the both *scEVR29* and the pTEF+intron -lip2pre-T4 lysozyme construct was transformed into *pah1* $\Delta$ strain (Fig. 6, column 4). Together, these results suggest that ER expansion in the *pah1* $\Delta$ strain may be necessary to support overexpression of *scEVR29*. This hypothesis is further supported by the similar mRNA expression measured in both strains (Fig. 7, columns 3,4) pointing to post-transcriptional differences. In addition, the secretion level of T4 lysozyme was changed insignificantly when *scERV29* was co-expressed under the weak promoter pTEF(star) in either po1f or *pah1* $\Delta$ strains (Fig. 6, column 5 and 6). Interestingly, the mRNA level of T4 lysozyme under weak promoter showed 1.5-fold increase in *pah1* $\Delta$ strain (Fig. 7, column 6, *P* value of < 0.01).

*STE13* encodes dipeptidyl aminopeptidase A which cleaves the signal peptide with recognition site X-Ala/X-Pro in Golgi [35]. It has been applied in the maturation of peptides and small molecule proteins in *S. cerevisiae* [36, 37]. In our study, *STE13* from *S. cerevisiae* (*scSTE13*) was also co-expressed with T4 lysozyme under both weak and strong promoter. The secretion level of T4 lysozyme was significantly decreased to 38.19 $\mu$ g/L/OD600 when it was co-expressed with *scSTE13* under strong promoter in po1f strain (Fig. 6, column 7, *P* value of < 0.0001). mRNA quantification indicates that the decrease in the secretion is accompanied by higher mRNA level. This was then alleviated with mRNA level decreases by more than 50% in *pah1* $\Delta$ strain (Fig. 7, lane 8). Similar observation on the negative correlation between secretion level and the mRNA level was found when *scSTE13* was co-expressed under a weak promoter in po1f strain where the secretion level returns from ~ 38.19 $\mu$ g/L/OD600 (Fig. 6, column 7) to ~ 291.53 $\mu$ g/L/OD600 (Fig. 6, column 9). A further improvement on the secretion level was observed when the *scSTE13* co-expression construct was transformed in *pah1* $\Delta$ strain (Fig. 6, column 10). Compared with the initially optimized construct, T4 lysozyme secretion yield was increased from ~ 300.78 to 349.23 $\mu$ g/L/OD600 when the dipeptidyl diaminopeptidase *scSTE13* was co-expressed under weak promoter in *pah1* $\Delta$ strain. At the same time, the expression level of T4 lysozyme remains the same between po1f and *pah1* $\Delta$ strains when expressed under weak promoter.

## Discussion

*Yarrowia lipolytica* has a large secretome, increased number of the plasma membrane SNARE complexes, and similarity with filamentous fungi cells all together make it a promising candidate for protein production [9]. In the native secretion signal lip2prepro, the pro sequence was regarded as unnecessary for the secretion but necessary for the folding of some serine protease-like proteins; however, removal of pro sequence from lip2prepro was reported to enhance the protein production of human interferon $\alpha$-2b [18]. In our study, T4 lysozyme secretion increased when the pro sequence was removed (Fig. 1, column 1 and 2). At the same time, the mRNA level decreased, which differs from the previous studies where the mRNA level was not affected by the pro sequence [18]. It has been reported that the secretion signal region could be serving as a nuclear export signal of an mRNA that lacks an intron. Deletion of the lip2 pro sequence within the context of the lip2pre-T4 lysozyme constructs might impair this function and reduce the mRNA export efficiency from nucleus to cytosol [38, 39]. Inclusion of the pTEF+intron sequence increases the gene expression level by threefold (Fig. 3, column 3), which is consistent with the prior reports [40]; however, the higher expression level was then reduced when either prepro or pre sequence was added. Some introns have been regarded as gene expression enhancers by enhancing the mRNA stability, facilitating mRNA export or increasing transcription initiation rates [40, 41], but its enhancement in mRNA export from nucleus could be compromised when 5’ untranslated regions (UTR) introns were in the presence of genes containing signal sequence coding region (SSCR) [39]. This incompatibility between 5’UTR introns and SSCR could explain the decrease of the gene expression level when intron was combined with secretion signal.

The generality of removing the pro sequence to enhance protein secretion is further supported by the secretion of $\alpha$-amylase (54.2 kDa) and GFP (27.3 kDa) in Fig. 3. Secretion of both proteins was improved significantly upon the removal of the pro sequence from the lip2prepro sequence. Interestingly the specific titer of both proteins was reduced to less than 30 $\mu$g/L/OD600. This change might hint that the secretion of proteins in *Y. lipolytica* could be size dependent and the secretion level of larger proteins ($\alpha$-amylase and GFP) is lower than smaller size protein (T4 lysozyme), which is in consistence with the observations on $\beta$-glycosidase secretion [42], or from some other factors relating to the folding and structure of the proteins.

ER expansion has been performed in several recent studies. The *pah1∆* enhanced membrane protein expression in *Y. lipolytica* and improved the membrane-associated enzymes for triterpene synthesis in *S. cerevisiae* [24, 33]*.* A study of secretion pathway engineering in *S. cerevisiae* directly showed that *pah1∆* was able to improve the secretion yield of heterologous proteins significantly by enhancing the level of trafficking vesicle formation and ER processing capability [26]. However, in our study, the *pah1∆* alone was unable to improve the secretion of T4 lysozyme. This suggests that under these conditions, the secretion of T4 lysozyme is not ER size or capacity limited.

In the study of secretory pathway engineering in *S. cerevisiae*, overexpression of *scERV29* under a strong promoter improved secretion of endoglucanase [26]; however, overexpression of *scERV29* decreased the secretion of $\alpha$-amylase in *S. cerevisiae* ER [43]. In this study, overexpression of *scERV29* under either a strong or weak promoter in *Y. lipolytica* po1f strain was unable to improve the secretion titer of T4 lysozyme (Fig. 6, column 3 and 5). Direct transformation of either the optimized vector or *ERV29* under the weak promoter into *pah1∆* strain did not exhibit significant improvement of T4 lysozyme secretion (Fig. 6, columns 1 and 2, columns 5 and 6). While expression of scERV29 from a strong promoter caused a decrease in T4 lysozyme secretion, expression of *scERV29* from a strong promoter in the *pah1∆* strain resulted nearly threefold improvement in T4 lysozyme secretion (Fig. 6, columns 3 and 4). This could be explained by ER stress induced by the *scERV29* overexpression counteracting the benefits on secretion, and relief of that ER stress by the *pah1∆* related ER expansion. It should be noted that there is a *Y. lipolytica* paralog of *ERV29* that has not been tested in the po1f or *pah1∆* strain [20].

*scSTE13* encodes diaminopeptidase to cleave the XA/XP recognition site in the secretion signal in Golgi compartment [44]. Overexpression of *scSTE13* to improve the secretion of proteins is seldom reported so far. Even though our strategy of using *STE13* with differing strength of promoters was unable to improve the yield of T4 lysozyme in po1f strain, it demonstrates overexpression of *STE13* under strong promoter impairs the protein production. This impairment can be alleviated when *STE13* was overexpressed under weak promoter (Fig. 6, column 7 and 9). This may imply that the weak coexpression of *scSTE13* is enough to process the secreted polypeptide while strong overexpression may decrease the processing efficiency. Overall, these results point to a striking difference in *Y. lipolytica* secretion compared to *S. cerevisiae*.

## Conclusions

In this study, gene expression and ER entry was first investigated. A 17-fold improvement in the secretion production of T4 lysozyme was achieved after the inclusion of intron and optimization of its combination with secretion signal. At the same time, the combination was found affecting the gene expression level. To achieve further increase in the protein production, ER and Golgi body were then focused. ER expansion has been reported improving the membrane protein yield in *Y. lipolytica* and enzyme secretion in *S. cerevisiae*. In our study, a direct transformation of the optimized construct into *pah1∆* strain was unable to improve the protein production significantly. Instead, similar increase in the secretion yield was observed when overexpression constructs of *scERV29* and *scSTE13* were transformed into *pah1∆* strain. Direct overexpression of the ER exit receptor *scERV29* and diamino peptidase *scSTE13* under both strong and weak promoter in po1f strain were unable to lead any improvement in secretion yield. A nearly threefold further improvement of the secretion yield was seen when *scERV29* was co-expressed under strong promoter in *pah1∆* strain. Our results hint that a different mechanism of secretion regulation might exist in ER and Golgi body in *Y. lipolytica*.

## Supplementary Information

**Additional file 1: Table S1.** Primers used for vector constructions and qPCR. **Table S2.** Synthesized gene sequences. **Table S3.** Summary of all the constructs secretion and mRNA level.

## Data Availability

The datasets during and/or analyzed during the current study available from the corresponding author on reasonable request.
