# Supplementary material for: Engineering heterologous enzyme secretion in Yarrowia lipolytica
Source: Microb Cell Fact. 2022 Jul 4;21:134. doi: 10.1186/s12934-022-01863-9 (PMC9252082; doi:10.1186/s12934-022-01863-9)
Supplement: Supplementary file 1 — Additional file 1: Table S1. Primers used for vector constructions and qPCR. Table S2. Synthesized gene sequences. Table S3. Summary of all the constructs secretion and mRNA level. [file 12934_2022_1863_MOESM1_ESM.docx]

Table S1 Primers used for vector constructions and qPCR

| Vectors | Primers | |
| --- | --- | --- |
|  | Forward | Reverse |
| PSL16-tef-intron-lip2prepro-T4 | TCATGTAATTAGTTATGTC | CCGCAGTTCTCCAGAAGCGA |
| PSL16-tef-intron-lip2pre-T4 | GCTGCCGCCCTCCCTTCCCCCATGAACATCTTCGAGATGC | AGATGTTCATGGGGGAAGGAGG |
| PSL16-tef-lip2pre-T4 | CCTTCTGAGTATAAGAATCATTCAAAATGAAATTTCCACCATCCTCTTC | TTTGAATGATTCTTATACTCAGAAGG |
| PSL16-tef-intron-T4 | CATCTCGAAGATGTTCATCTGCGGTTAGTACTGC | GCAGTACTAACCGCAGATGAACATCTTCGAGATGC |
| PSL16-tef-intron-lip2pre-T4-B1-tef-intron-Ste13 | CTTTTTGCAGTACTAACCGCAGATGTCTGCTTCAACTCATTC | AACTAATTACATGAGGCTAGCTTACAGCTCGTCTCGTAAATGCAAAACTTCAGTGTTG |
|  | ATCATGGTCATAGCTGTTTCCTG | GACGCTCGAAGGCTTTAATTTGCGCGATCGCCGTATTACGTAGATCCAGACAGGCGCGCC |
|  | CTTTTTGCAGTACTAACCGCAGATGTCTGCTTCAACTCATTC | AACTAATTACATGAGGCTAGCTTACAGCTCGTCTCGTAAATGCAAAACTTCAGTGTTG |
|  | GCTAGCCTCATGTAATTAG | CTGCGGTTAGTACTGC |
| PSL16-tef-intron-lip2pre-T4-B1-tef(star)-Ste13 | CTTTTTGCAGTACTAACCGCAGATGTCTGCTTCAACTCATTC | AACTAATTACATGAGGCTAGCTTACAGCTCGTCTCGTAAATGCAAAACTTCAGTGTTG |
|  | ATCATGGTCATAGCTGTTTCCTG | GACGCTCGAAGGCTTTAATTTGCGCGATCGCCGTATTACGTAGATCCAGACAGGCGCGCC |
|  | AGTATAAGAATCATTCAAAATGATGTCTGCTTCAACTCATTC | AACTAATTACATGAGGCTAGCTTACAGCTCGTCTCGTAAATGCAAAACTTCAGTGTTG |
|  | GCTAGCCTCATGTAATTAG | CATTTTGAATGATTCTTATAC |
| PSL16-tef-intron-lip2pre-GFP | GCCGCCCTCCCTTCCCCCATGGTGAGCAAGCAGATC | GGTGATGGTGATGGTGACTACCCACCCACTCGTGCAG |
|  | GGTAGTCACCATCACCATCACC | GGGGAAGGAGGCGGC |
| PSL16-tef-intron-lip2prepro-GFP | CGCAGTTCTCCAGAAGCGAATGGTGAGCAAGCAG | GGTGATGGTGATGGTGACTACCCACCCACTCGTGCAG |
|  | GGTAGTCACCATCACCATCACC | TCGCTTCTGGAGAACTGCG |
| PSL16-tef-intron-lip2pre-$\alpha$- Amylase | GGTAGTCACCATCACCATCACC | GGGGGAAGGAGGGCGGC |
| PSL16-tef-intron-lip2prepro-$\alpha$-Amylase | CGCAGTTCTCCAGAAGCGAGAGAAGGAAGAGCGAACC | TTAATGGTGATGGTGATGGTGACTAC |
|  | GGTAGTCACCATCACCATCACC | GGGGGAAGGAGGGCGGC |
| PARENT_nsiI_Crispr_PAH1_Leu | CCACACAACCACACGATG | GGCTTACTTGCCCCCAA |
| qPCR T4 lysozyme | AAGGACACCGAGGGTTACTA | TGGTAATCACGCCGTTACAG |
| Beta-actin | AAAGACCTCTATGCCAACACA | GCAGTGATCTCCTTCTGCATC |

Table S2 Synthesized gene sequences

| Gene | Sequence |
| --- | --- |
| T4 lysozyme | ATGAACATCTTCGAGATGCTGCGAATTGACGAGGGACTGCGACTGAAGATCTACAAGGACACCGAGGGTTACTACACCATCGGCATTGGACACCTGCTGACCAAGTCTCCCTCCCTGAACGCCGCTAAGTCTGAGCTGGACAAGGCCATCGGTCGAAACTGTAACGGCGTGATTACCAAGGACGAGGCTGAGAAGCTGTTCAACCAGGACGTGGACGCTGCTGTCCGAGGTATCCTGCGAAACGCCAAGCTGAAGCCTGTGTACGACTCCCTGGACGCCGTCCGACGATGCGCTCTGATTAACATGGTGTTCCAGATGGGAGAGACCGGCGTCGCTGGATTCACCAACTCTCTGCGAATGCTGCAGCAGAAGCGATGGGACGAGGCCGCTGTGAACCTGGCCAAGTCCCGATGGTACAACCAGACCCCCAACCGAGCTAAGCGAGTCATTACCACCTTCCGAACCGGAACCTGGGACGCCTACAAGAACCTG |
| T4 lysozyme amino acid sequence | MNIFEMLRIDEGLRLKIYKDTEGYYTIGIGHLLTKSPSLNAAKSELDKAIGRNCNGVITKDEAEKLFNQDVDAAVRGILRNAKLKPVYDSLDAVRRCALINMVFQMGETGVAGFTNSLRMLQQKRWDEAAVNLAKSRWYNQTPNRAKRVITTFRTGTWDAYKNLGSHHHHHH |
| hrGFP | ATGGTGAGCAAGCAGATCCTGAAGAACACCGGCCTGCAGGAGATCATGAGCTTCAAGGTGAACCTGGAGGGCGTGGTGAACAACCACGTGTTCACCATGGAGGGCTGCGGCAAGGGCAACATCCTGTTCGGCAACCAGCTGGTGCAGATCCGCGTGACCAAGGGCGCCCCCCTGCCCTTCGCCTTCGACATCCTGAGCCCCGCCTTCCAGTACGGCAACCGCACCTTCACCAAGTACCCCGAGGACATCAGCGACTTCTTCATCCAGAGCTTCCCCGCCGGCTTCGTGTACGAGCGCACCCTGCGCTACGAGGACGGCGGCCTGGTGGAGATCCGCAGCGACATCAACCTGATCGAGGAGATGTTCGTGTACCGCGTGGAGTACAAGGGCCGCAACTTCCCCAACGACGGCCCCGTGATGAAGAAGACCATCACCGGCCTGCAGCCCAGCTTCGAGGTGGTGTACATGAACGACGGCGTGCTGGTGGGCCAGGTGATCCTGGTGTACCGCCTGAACAGCGGCAAGTTCTACAGCTGCCACATGCGCACCCTGATGAAGAGCAAGGGCGTGGTGAAGGACTTCCCCGAGTACCACTTCATCCAGCACCGCCTGGAGAAGACCTACGTGGAGGACGGCGGCTTCGTGGAGCAGCACGAGACCGCCATCGCCCAGCTGACCAGCCTGGGCAAGCCCCTGGGCAGCCTGCACGAGTGGGTG |
| hrGFP amino acid sequence | MVSKQILKNTGLQEIMSFKVNLEGVVNNHVFTMEGCGKGNILFGNQLVQIRVTKGAPLPFAFDILSPAFQYGNRTFTKYPEDISDFFIQSFPAGFVYERTLRYEDGGLVEIRSDINLIEEMFVYRVEYKGRNFPNDGPVMKKTITGLQPSFEVVYMNDGVLVGQVILVYRLNSGKFYSCHMRTLMKSKGVVKDFPEYHFIQHRLEKTYVEDGGFVEQHETAIAQLTSLGKPLGSLHEWVGSHHHHHH |
| $\alpha$-amylase | GAGAAGGAAGAGCGAACCTGGCAGGACGAGGCCATCTACTTCATTATGGTGGACCGATTCAACAACATGGACCCCACCAACGACCAGAACGTCAACGTGAACGACCCCAAGGGATACTTCGGCGGTGACCTCAAGGGCGTGACCGCCAAGCTGGACTACATCAAGGAGATGGGCTTCACCGCTATCTGGCTGACCCCCATTTTCAAGAACATGCCCGGAGGCTACCACGGTTACTGGATTGAGGACTTCTACCAGGTGGACCCCCACTTCGGCACCCTGGGTGACCTCAAGACCCTGGTCAAGGAAGCCCACAAGCGAGACATGAAGGTCATCCTGGACTTCGTCGCTAACCACGTGGGTTACAACCACCCCTGGCTGCACGACCCCACCAAGAAGGACTGGTTCCACCCCAAGAAGGAGATTTTCGACTGGAACGACCAGACCCAGCTGGAGAACGGATGGGTCTACGGACTCCCCGACCTGGCTCAGGAGAACCCCGAGGTGAAGACCTACCTCATCGACGCCGCTAAGTGGTGGATCAAGGAGACCGACATTGACGGCTACCGACTGGACACCGTCCGACACGTGCCCAAGTCCTTCTGGCAGGAGTTCGCTAAGGAAGTCAAGTCTGTGAAGAAGGACTTCTTCCTGCTGGGAGAAGTGTGGTCTGACGACCCTCGATACATCGCCGACTACGGAAAGTACGGCATTGACGGTTTCGTGGACTACCCCCTCTACGGTGCTGTGAAGCAGTCCCTGGCTCGACGAGACGCTTCTCTCCGACCCCTGTACGACGTCTGGGAGTACAACAAGACCTTCTACGACCGACCCTACCTGCTCGGTTCCTTCCTCGACAACCACGACACCGTGCGATTCACCAAGCTGGCCATCGACAACCGAAACAACCCCATCTCTCGAATTAAGCTCGCCATGACCTACCTGTTCACCGCTCCCGGCATCCCCATTATGTACTACGGCACCGAGATCGCCATGAACGGTGGACAGGACCCCGACAACCGACGACTCATGGACTTCCGAGCTGACCCCGAGATCATTGACTACCTCAAGAAGATTGGACCTCTGCGACAGGAGCTCCCCTCCCTGCGACGAGGTGACTTCACCCTGCTCTACGAGAAGGACGGAATGGCCGTCCTGAAGCGACAGTACCAGGACGAGACCACCGTGATCGCTATTAACAACACCTCTGAGACCCAGCACGTCCACCTCACCAACGACCAGCTGCCCAAGAACAAGGAGCTCCGAGGATTCCTGCTCGACGACCTGGTGCGAGGTGACGAGGACGGATACGACCTGGTCCTCGACCGAGAGACCGCTGAGGTGTACAAGCTGCGAGAGAAGACC |
| $\alpha$-amylase amino acid sequence | EKEERTWQDEAIYFIMVDRFNNMDPTNDQNVNVNDPKGYFGGDLKGVTAKLDYIKEMGFTAIWLTPIFKNMPGGYHGYWIEDFYQVDPHFGTLGDLKTLVKEAHKRDMKVILDFVANHVGYNHPWLHDPTKKDWFHPKKEIFDWNDQTQLENGWVYGLPDLAQENPEVKTYLIDAAKWWIKETDIDGYRLDTVRHVPKSFWQEFAKEVKSVKKDFFLLGEVWSDDPRYIADYGKYGIDGFVDYPLYGAVKQSLARRDASLRPLYDVWEYNKTFYDRPYLLGSFLDNHDTVRFTKLAIDNRNNPISRIKLAMTYLFTAPGIPIMYYGTEIAMNGGQDPDNRRLMDFRADPEIIDYLKKIGPLRQELPSLRRGDFTLLYEKDGMAVLKRQYQDETTVIAINNTSETQHVHLTNDQLPKNKELRGFLLDDLVRGDEDGYDLVLDRETAEVYKLREKTGSHHHHHH |
| Pre | AAACTTTCCACCATCCTCTTCACAGCCTGCGCTACCCTGGCTGCCGCCCTCCCTTCCCCC |
| Pro | ATCACTCCTTCTGAGGCCGCAGTTCTCCAGAAGCGA |
| TEF | AGACCGGGTTGGCGGCGCATTTGTGTCCCAAAAAACAGCCCCAATTGCCCCAATTGACCCCAAATTGACCCAGTAGCGGGCCCAACCCCGGCGAGAGCCCCCTTCTCCCCACATATCAAACCTCCCCCGGTTCCCACACTTGCCGTTAAGGGCGTAGGGTACTGCAGTCTGGAATCTACGCTTGTTCAGACTTTGTACTAGTTTCTTTGTCTGGCCATCCGGGTAACCCATGCCGGACGCAAAATAGACTACTGAAAATTTTTTTGCTTTGTGGTTGGGACTTTAGCCAAGGGTATAAAAGACCACCGTCCCCGAATTACCTTTCCTCTTCTTTTCTCTCTCTCCTTGTCAACTCACACCCGAAATCGTTAAGCATTTCCTTCTGAGTATAAGAATCATTCAAA |
| Intron | GTGAGTTTCAGAGGCAGCAGCAATTGCCACGGGCTTTGAGCACACGGCCGGGTGTGGTCCCATTCCCATCGACACAAGACGCCACGTCATCCGACCAGCACTTTTTGCAGTACTAACCGCAG |

Table S3 Summary of all the constructs secretion and mRNA level

| Genotype | Concentration($\mu$g/OD600/L) | Expression level |
| --- | --- | --- |
| Psl16-tef-prepro-T4 | 18.2$\pm$5.4 | 1.3$\pm$0.2 |
| Psl16-tef-pre-T4 | 73.5$\pm$5.1 | 0.6$\pm$0.1 |
| Psl16-tef-intron-T4 | 183.0$\pm$35.6 | 3.7$\pm$0.7 |
| Psl16-tef-intron-prepro-T4 | 157.5$\pm$15.5 | 1.0$\pm$0.4 |
| Psl16-tef-intron-lip2pre-T4 | 300.8$\pm$42.9 | 1.9$\pm$0.5 |
| Psl16-tef-intron-lip2prepro-GFP | 5.3$\pm$0.9 | Not test |
| Psl16-tef-intron-lip2pre-GFP | 9.5$\pm$1.2 | Not test |
| Psl16-tef-intron-lip2prepro-$\alpha$-amylase | 6.1$\pm$2.3 | Not test |
| Psl16-tef-intron-lip2pre-$\alpha$-amylase | 21.7$\pm$2.7 | Not test |
| Psl16-tef-intron-lip2pre-T4-$\Delta pah1$ | 284.3$\pm$35.4 | 0.4$\pm$0.0 |
| Psl16-tef-intron-lip2pre-T4-psl16-tef-intron-*scERV29* | 206.7$\pm$36.8 | 0.7$\pm$0.2 |
| Psl16-tef-intron-lip2pre-T4-psl16-tef-intron-*scERV29*-$\Delta pah1$ | 842.5$\pm$147.9 | 1.2$\pm$0.3 |
| Psl16-tef-intron-lip2pre-T4-psl16-tef-star-*scERV29* | 307.7$\pm$9.9 | 1.0$\pm$0.3 |
| Psl16-tef-intron-lip2pre-T4-psl16-tef-star-*scERV29*-$\Delta pah1$ | 303.4$\pm$11.1 | 2.5$\pm$0.2 |
| Psl16-tef-intron-lip2pre-T4-psl16-tef-intron-*scSTE13* | 38.2$\pm$3.2 | 3.6$\pm$0.4 |
| Psl16-tef-intron-lip2pre-T4-psl16-tef-intron-*scSTE13*-$\Delta pah1$ | 107.4$\pm$6.6 | 1.6$\pm$0.2 |
| Psl16-tef-intron-lip2pre-T4-psl16-tef-star-*scSTE13* | 291.5$\pm$13.2 | 1.8$\pm$0.5 |
| Psl16-tef-intron-lip2pre-T4-psl16-tef-star-*scSTE13*-$\Delta pah1$ | 349.2$\pm$9.0 | 1.6$\pm$0.3 |
